# Molecular Dynamics Simulation on the Interfacial Behavior of Over-Molded Hybrid Fiber Reinforced Thermoplastic Composites

**DOI:** 10.3390/polym12061270

**Published:** 2020-06-02

**Authors:** Bingyan Jiang, Muhan Zhang, Liang Fu, Mingyong Zhou, Zhanyu Zhai

**Affiliations:** 1 State Key Laboratory of High Performance and Complex Manufacturing, Light Alloy Research Institute, Central South University, Lushan South Road 932, Changsha 410083, China; jby@csu.edu.cn (B.J.); 183811004@csu.edu.cn (M.Z.); 2 College of Mechanical and Electrical Engineering, Central South University, Lushan South Road 932, Changsha 410083, China; fuliangcsu@csu.edu.cn (L.F.); mingyong.zhou1989@csu.edu.cn (M.Z.)

**Keywords:** thermoplastic composite over-molding, hybrid thermoplastic composite, molecular dynamics simulation, heterogeneous interface, adhesion

## Abstract

Hybrid fiber reinforced thermoplastic composites are receiving important attention in lightweight applications. The fabrication process of hybrid thermoplastic composites is that discontinuous fiber reinforced thermoplastics are injected onto the continuous fiber reinforced thermoplastics by over-molding techniques. The key issue during this process is to get a reliable interfacial bonding strength. To understand the bonding mechanism at the heterogeneous interface of hybrid thermoplastic composites which is difficult to obtain through experimental investigations, a series of molecular dynamic (MD) simulations were conducted in this paper. The influence of processing parameters on the interfacial characteristics, i.e., the distribution of interfacial high-density enrichment areas, radius of gyration, diffusion coefficient and interfacial energy, were investigated during the forming process of a heterogeneous interface. Simulation results reveal that some of molecule chains get across the interface and tangle with the molecules from the other layer, resulting in the penetration phenomenon near the interface zone. In addition, the melting temperature and injection pressure exhibit positive effects on the interfacial properties of hybrid composites. To further investigate the interfacial bonding strength and fracture mechanism of the heterogeneous interface, the uniaxial tensile and sliding simulations were performed. Results show that the non-bonded interaction energy plays a crucial role during the fracture process of heterogeneous interface. Meanwhile, the failure mode of the heterogeneous interface was demonstrated to evolve with the processing parameters.

## 1. Introduction

A thermoplastic composite over-molding (TCO) process involves the combination of stamping and injection molding techniques and enables the production of hybrid fiber reinforced thermoplastic composites (hybrid composites) structures in a single economic process stage of molding [1,2]. The continuous fiber reinforced thermoplastic composites (CFRT) in the form of laminate (so called organosheet) is generally chosen as a base of hybrid composite structures [3,4]. For the TCO process of hybrid composites, organosheet is firstly thermoformed and subsequently over-molded with pure thermoplastic or short fiber reinforced thermoplastic (SFRT) in an injection mold. Accordingly, the interface between the substructures (i.e., organosheet and SFRT) is developed. As a load-carrying region, the interface bonding strength is assumed as a key property for hybrid composite parts because low interfacial properties tend to result in an interfacial failure and subsequent structure failure [1,5] of the bulk composites.

In order to exploit the lightweight potential of hybrid composites structures, the interface between the individual components has been intensely studied [2,6,7]. Fiorotto et al. [7] carried out three-point flexure tests on hybrid composites to evaluate the bonding quality between the over-molded ribs and CFRT. The results show that the melting temperature has an obvious effect on the bonding strength. Moreover, a good interface of hybrid composites could be achieved when the preheating temperature of CFRT is higher than the melting temperature of the matrix. Tanaka et al. [6] studied the effect of mold temperature on the bonding strength between the over-molded part and CFRT via T-shape tensile tests. They found that the penetrated height of continuous fiber into a rib becomes higher with increasing the mold temperature, which contributes to a higher interfacial strength of hybrid composites. Joppich et al. [8] investigated the bonding strength of manufactured ribbed plates via quasi-static rib pull-off tests and analyzed the process-induced features. The obtained results demonstrated that the bonding strength between the over-molded rib and CFRT is primarily affected by the preheating temperature of CFRT. According to the above literatures, one can arrive at a general conclusion that the processing parameters have an important influence on the quality and bonding strength between the over-molded part and CFRT in the hybrid composites.

Regarding the forming process of interface in hybrid composites, it can be found that two essential sequences of the intimate contact and the self-diffusion of molten polymers at the interface are proceed. However, it is difficult to identify the detailed information about the interface adhesion mechanism through macroscopic mechanical behavior. Molecular dynamics (MD) simulation is a method to obtain the interfacial behavior and properties of materials at the atomic or molecular scale level, which can provide detailed insights into molecular diffusion and interactions across the interface caused by chemical bonding. It has been used to determine the properties of metal/metal [9,10], polymer/metal [11,12,13,14,15,16,17], polymer/ceramics [18,19], polymer/fiber [20,21,22,23,24], polymer/polymer [25,26,27,28] and even polymer/nanoparticle [29] interfaces. In particular, Wang et al. [25] investigated the interfacial energy of hybrid composites through MD methods. They claimed that the interfacial energy was generated mainly from van der Waals forces and electrostatic interaction. However, this work still has some merits to be further studied and supplemented, for example, molecular diffusion and interfacial characteristics were not involved in the work. Despite all these efforts, to date, the understanding of the interfacial bonding mechanism in the TCO fabrication of heterogeneous hybrid thermoplastic composites is still somewhat limited or lacking.

In this study, continuous glass fiber reinforced polypropylene (CFR-PP) was chosen as a base of hybrid composites. Short glass fiber reinforced polyamide 66 (SFR-PA66) was injected on CFR-PP by over-molding technique. A molecular model involving PA66/PP hybrid system was constructed to investigate the interface bonding mechanism of hybrid composites using MD simulations. The forming process of SFR-PA66/CFR-PP heterogeneous interface during TCO process was firstly studied. Meanwhile, the influence of melting temperature and injection pressure on interfacial bonding behavior associated with the radius of gyration, diffusion coefficient and interfacial energy were investigated comparatively. Finally, the uniaxial tensile and sliding simulations were carried out to analyze the interfacial bonding strength and fracture mechanism. The obtained results will serve as a useful reference in terms of the determination of TCO processing parameters and provide an understanding about the bonding mechanism and fracture mechanism of hybrid composites.

## 2. Simulation Model and Methodology

### 2.1. Model Construction

To investigate the bonding behavior of SFR-PA66 and CFR-PP parts during TCO process, the atomistic model of SFR-PA66/CFR-PP interface was constructed. It is well known that the matrix layers are positioned at the outer layer of both SFR-PA66 and CFR-PP, thus, the atomistic model of interface between SFR-PA66 and CFR-PP was built simply as that of PA66/PP interface. Moreover, the bonding between SFR-PA66 and CFR-PP parts is assumed to complete during injection process, without considering the subsequent stages, such as holding pressure and cooling stages.

**Figure 1:**
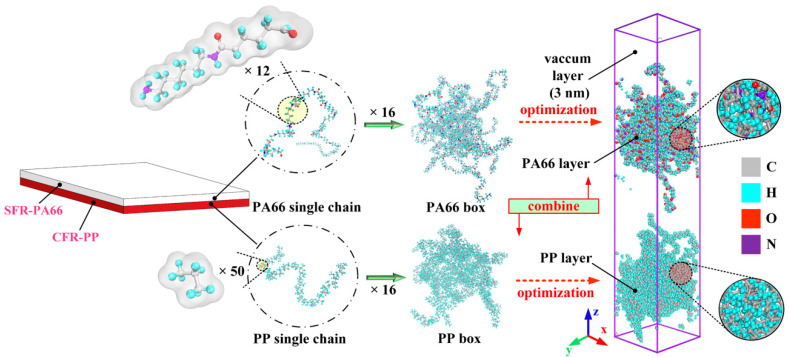
Conformations of all-atom model of hybrid PA66–PP system.

Before assembling the injection over-molded hybrid system, the atomistic models of PP and PA66 layers were separately constructed. Some important modeling parameters, including the degree of polymerization, number of chains, initial density, and box size, are given in Table 1. It can be found that the degree of polymerization chosen in this simulation was relatively low compared with the real materials, which is attributed to nanometer scale in the model [28]. The limitation of this model is that the lower degree of polymerization may affect the prediction accuracy of the mechanical properties of the interface. However, due to the error compensation, the interfacial structure and mechanical properties can be predicted by calculation in a shorter time [30]. The all-atom composite model is composed of the PA66 model and PP model, as shown in Figure 1. Since the initial conformation of PP and PA66 models were unstable, some optimizations were required before assembling the hybrid system. To be specific, both PA66 and PP models were relaxed by energy minimization using the steepest descent, the conjugate gradient and Newton methods with 10,000 iterations, respectively.

### 2.2. Simulation Procedure

In this simulation, the influence of melting temperature and injection pressure on the interfacial bonding behavior between PA66 and PP layers was mainly investigated. The intermolecular and non-bonded interactions between atoms were described by the consistent valence force field (CVFF), which has been gradually applied to a variety of polypeptides, proteins and a large number of organic molecular systems and broadly applied to investigate the interfacial properties of polymer systems [11,12,28]. In this work, the force field consists of not only bond stretching, angular bending, torsion and improper potentials, but also non-bonded interactions, including Lennard-Jones (12–6) and Coulomb potentials. The energy function of CVFF is shown as Equation (1):

(1) $$\begin{array}{cc} E & =E_{bond}+E_{angle}+E_{torsion}+E_{improper}+E_{vdw}+E_{coul}\\ & =\sum_{bond}K_{b}{\left(b-b_{0}\right)}^{2}+\sum_{angle}K_{a}{\left(\theta -\theta_{0}\right)}^{2}+\sum_{torsion}K_{t}\left[1+s\cos\left(n\emptyset_{t}\right)\right]\\ & +\sum_{improper}K_{o}\left[1+s\cos\left(n\emptyset_{i}\right)\right]+\sum_{ij}4\varepsilon_{ij}\left[{\left(\frac{\sigma_{ij}}{r}\right)}^{12}-{\left(\frac{\sigma_{ij}}{r}\right)}^{6}\right]+\sum_{ij}\frac{q_{i}q_{j}}{\varepsilon r_{ij}} \end{array}$$

where $E_{bond}$ is the functional term of bond stretching. $E_{angle}$ and $E_{torsion}$ are the angle bending and dihedral angle torsion, respectively. $E_{improper}$ is the improper of the bonded interaction. $E_{vdw}$ and $E_{coul}$ are the van der Waals and Coulombic, respectively. The *K*_b_, *K*_a_, *K*_t_ and *ε* are constants of the bond stretching potential, angular bending potential, torsion potential and non-bonded interaction, respectively.

**Figure 2:**
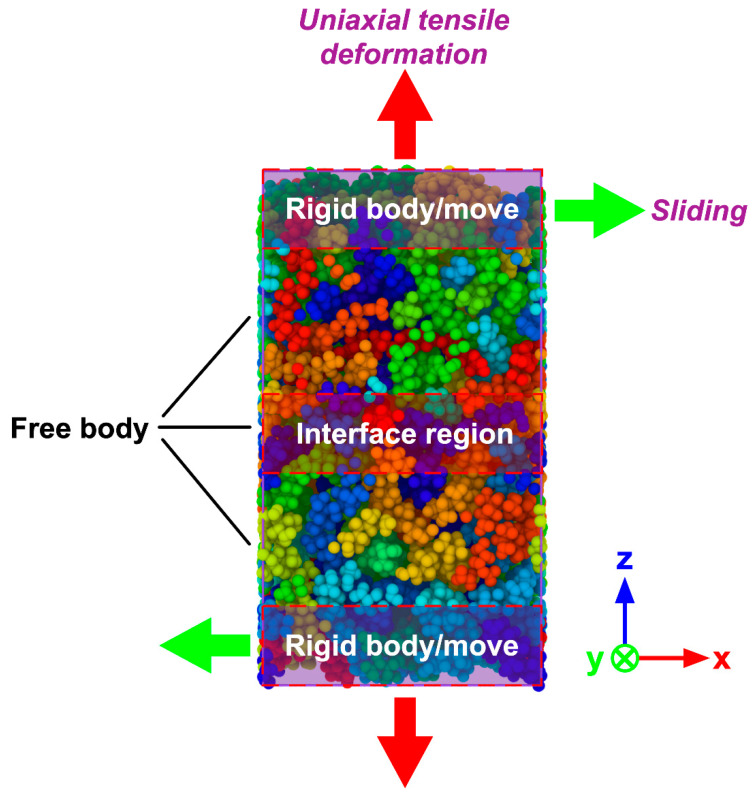
Molecular model of hybrid PA66–PP system for uniaxial tensile and sliding deformation.

At the beginning of the simulations, PP layer was preheated with 100 ps (∆*t* = 1 fs) to reach the temperature of 180 °C, resulting in a warm/warm contact between the PA66 and PP layers, which can improve the bonding quality. The PA66 layer was heated up to the targeted melting temperatures (*T*_m_) for also 100 ps (∆*t* = 1 fs) listed in Table 2, respectively, and then stayed with the targeted temperature in a fixed number of atoms N, volume V, and temperature T (NVT). After that, different pressures were applied to the hybrid system separately. It should be noticed that the preheating temperature of PP layer was always kept at 180 °C for all the simulation tasks. When the melting temperature was varied in the range of from 250 to 310 °C, the injection pressure was kept as 4 MPa. The melting temperature was set as 280 °C when varying injection pressure. The detailed processing parameters can be found in Table 2. In the present work, the total simulation time was set as 300 ps (∆*t* = 1 fs). The periodic boundary conditions (PBC) were applied in the x- and y- axis to replicate the large aspect ratio of polymer particles, while the free boundary conditions were applied to the z- axis so that the whole system can be effectively compressed during the injection process. After the simulation of TCO process, to further explore the mechanical properties and fracture mechanism of the PA66–PP interface, the uniaxial tensile deformation and sliding deformation were performed. During mechanical separation simulations, the atoms of the top layer from PA66 as well as the atoms of the bottom layer from PP were handled as rigid bodies while keeping the middle part unconstrained, as shown in Figure 2. Tensile loading was simultaneously applied for the top/bottom rigid body with a velocity of 0.05 nm/ps along the +z/-z direction under a constant NVT ensemble with 300K. To achieve the interfacial shear behavior, the relative displacement was loaded on the two rigid bodies with the velocity of 0.05 nm/ps, as shown in Figure 2. The loading direction was parallel to the x-y plane. Verlet velocity algorithm was used to integrate Newtown’s equation of motions. Besides, Nosé–Hoover method was employed to act as thermostat during the whole process. In addition, the tensile stress was applied once per 2000 time steps and calculated using the Virial theorem throughout only the middle zone. All the simulation procedures discussed were performed in a computer cluster by LAMMPS 64-bit version (16Mar2018-MPI), an open-source molecular dynamics package distributed by Sandia National Laboratories, Livermore, CA, USA [31].

## 3. Results and Discussion

### 3.1. PA66–PP Interface Forming Process

**Figure 3:**
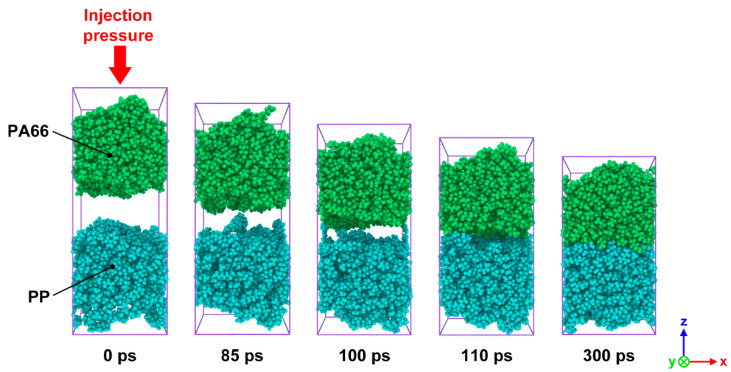
Snapshots of the bonding process of SFR-PA66 and CFR-PP under the processing parameters of *T*_p_ = 180 °C, *T*_m_ = 280 °C and *P* = 4 MPa at simulation times of 0, 85, 100, 110 and 300 ps.

In order to observe the intermolecular diffusion across the interface and to allow for a clear view of the interface forming process, PP and PA66 layers were painted in different colors, as shown in Figure 3. It can be seen that the whole system along z-axis was significantly compressed caused by injection pressure. There is an obvious gap between PA66 and PP layers at the initial stage of injection process. As the simulation proceeds, the gap gradually disappears. When the shortest distance between atoms from two layers was less than the cut-off distance of 1.25 nm, the mutual attraction of molecules would be accelerated due to chemical bonds. It finally results in a stable interfacial phase, which is a region stacked by a large number of molecules. It is worth noting that polymer molecules near the interfacial zone get across the interface and penetrate to the other layer, resulting in a series of interlocking nanostructures. The appearance of these nanostructures also indicates that a mechanical interlock effect contributes to the formation of heterogeneous interface of hybrid composites.

**Figure 4:**
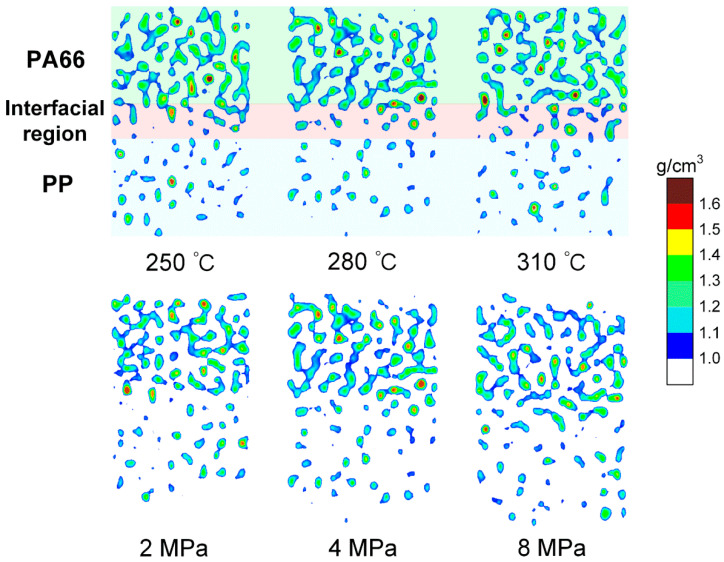
Nephogram of the high-density concentration of composite system along the z direction at 300 ps under different processing parameters.

Figure 4 shows the nephogram of high-density concentration area of PA66–PP hybrid system at 300 ps under different processing parameters. It is clearly visible that there is a density transition region in the PA66–PP interface system regardless of melting temperature and injection pressure. The transition region is composed of atomic mixing from PA66 and PP layers, which further demonstrates that the formation of the interfacial region depends on both self- and inter-diffusions. Moreover, increasing melting temperature (310 °C) or injection pressure (8 MPa) would favor the diffusion of atoms near the interface, thus the high-density distribution area evolves from scattered dots to denser patches.

### 3.2. Effect of Processing Parameters on Bonding Behavior

#### 3.2.1. Radius of Gyration

During the TCO process, the formation of the PA66–PP interface depends on not only on the mutual movement of two-phase molecules, but also on the self-movement of molecules. In order to further investigate the mechanism of molecules’ self-motion, the radius of gyration ($R_{g}$) of polymer molecules was studied. It is a measure to characterize the size of polymer macromolecules regarding their center-of-mass. It can be approximated as Equation (2):

(2) $$R_{g}=\sqrt{\frac{1}{M}\underset{i}{\sum }m_{i}{\left(r_{i}-r_{cm}\right)}^{2}}$$

where *M* and *r*_cm_ are the total mass of the polymer chains in the simulation system and the center-of-mass position of the chains, respectively. It is assumed that a plurality of chain units is contained in a polymer chain. The mass of each chain unit is *m*_i_ and *r*_i_ is the distance of an atom to the center-of-mass position of a single chain. Generally, a small value of $R_{g}$ corresponds to the curling and shrinking of molecular chains.

**Figure 5:**
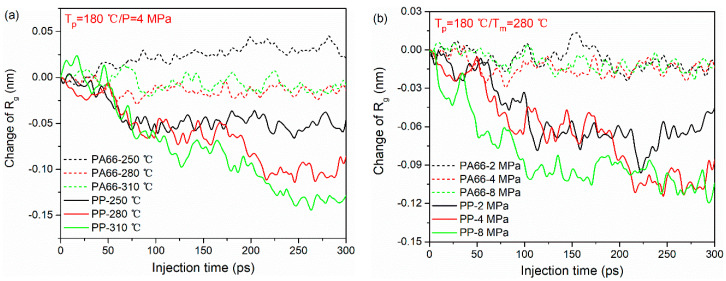
Radius of gyration of PA66 and PP during bonding process under different processing parameters: (**a**) melting temperatures and (**b**) injection pressure.

The variations of $R_{g}$ (characterized as the difference between the initial and final values of $R_{g}$ during TOC process) in PA66 and PP layers under various processing parameters are shown in Figure 5. As can be seen, under the same processing condition, the changes of $R_{g}$ in PP molecular chains are larger than that of the PA66 molecular chains for all the cases. This means that during the TCO process, PP chains at the interface are more likely to curl due to its lower stiffness and shorter chain length. Meanwhile, it can also be observed that the increase in melting temperature and injection pressure can play a positive role in the entanglement of PP chains. Figure 5a shows the influence of melting temperature on the $R_{g}$ of PA66 and PP layers. Clearly, the change of $R_{g}$ in PP chains at the melting temperature of 310 °C is higher than other cases. This behavior indicates that the molecules move easily because the internal friction among macromolecules becomes weak at higher melting temperature. With the increase in injection pressure, the changes of $R_{g}$ in PP chains decreases gradually, as shown in Figure 5b. Accordingly, it can be understood that the higher the external stress loaded, the larger the deformation of polymer molecular chains is. However, this phenomenon is not obvious in the PA66 layer. Here, the authors speculate that this may be related to the higher rigidity of the PA66 molecule and the limitations of the model size.

#### 3.2.2. Interfacial Bonding Energy and Diffusion Coefficient

To investigate the bonding mechanism of PA66 and PP layers during TCO process, the diffusion coefficients under different processing parameters were studied.

According to the fluctuation-dissipation theory of non-equilibrium thermodynamics [32], the total mean square displacement (MSD) of polymers can be obtained through Equation (3). Here, all the atoms from PA66 and PP layers were considered since the size of all-atom model of PA66–PP composites is relatively small. The Diffusion coefficient *D* was calculated from the linear part of MSD-*t* curve by following the Einstein relation, as shown in Equations (3–4).

(3) $$\mathrm{MSD}=6t\langle {\left|r_{i}\left(t\right)-r_{i}\left(0\right)\right|}^{2}\rangle$$

(4) $$\;D=\underset{t\to \infty }{\lim}\frac{1}{6t}\langle {\left|r_{i}\left(t\right)-r_{i}\left(0\right)\right|}^{2}\rangle$$

where *r*_i_(*t*) and *r*_i_(0) represent the position vectors of the first atom at time *t* and zero, respectively.

**Figure 6:**
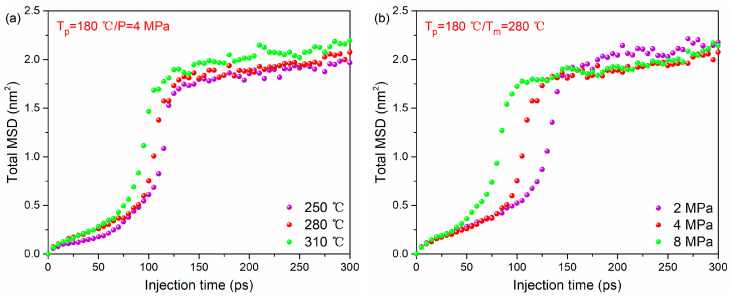
The MSD–time curve of PA66–PP hybrid system under various processing parameters: (**a**) melting temperatures and (**b**) injection pressure.

The total MSD–time curves of the whole system during bonding process under different melting temperatures and injection pressures are given in Figure 6a,b (the MSDs for each thermoplastic system are given in Figure S1). It can be found that at a higher melting temperature or injection pressure, the total MSD tends to increase with time rapidly. This illustrates that increasing melting temperature or injection pressure can accelerate the movement of polymer molecules. Therefore, the order of MSD at different temperatures and injection pressure is the same as that of a high-density concentration.

**Figure 7:**
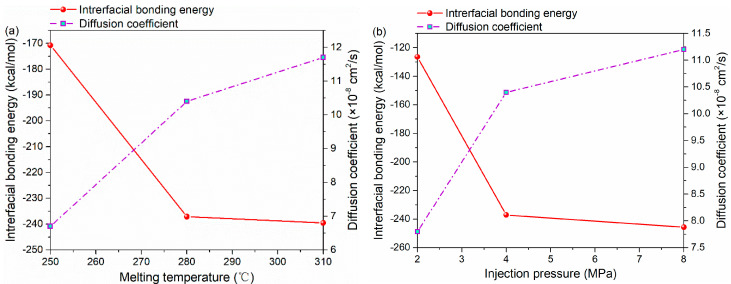
The interfacial bonding energy and diffusion coefficient of PA66–PP hybrid system under various processing parameters: (**a**) melting temperatures and (**b**) injection pressure.

The interfacial bonding energy and diffusion coefficient under different processing parameters are shown in Figure 7. By increasing the melting temperature from 250 to 310 °C, the diffusion coefficient increases from 6.7 × 10^−8^ cm^2^/s to the maximum value of 11.7 × 10^−8^ cm^2^/s. Meanwhile, there is also a positive correlation between injection pressure and diffusion coefficient. The diffusion coefficient under the injection pressure of 8 MPa is 43.6% higher than that under 2 MPa, as shown in Figure 7b.

The Chemical bonding theory [33] was applied to study the interface of composite materials in order to explore how the interaction between molecules affects the formation of the interface. The negative value of the interaction energy between the PA66 and PP layers is the interfacial energy of the system. The interface energy serves as a rule to evaluate the strength of interfacial bonding. The larger the energy, the more work is needed to destroy the interface and the more stable the structure will be. The interfacial energy was calculated by the following Equation (5):

*E*_*interfacial*_ = *E*_*inter*_ = *E*_*system*_ − (*E*_*PP*_ + *E*_*PA66*_) (5)

where *E_interfacial_* is the energy of the bonding interface, while *E_system_*, *E_PP_* and *E_PA66_* are the energies of the whole system, PP layer and PA66 layer, respectively.

Figure 7a illustrates the variation in the interfacial energy of the PA66–PP interface with melting temperature. As seen, the interfacial energy increases (absolute value) steeply from 170.76 to 239.54 kcal/mol when the melting temperature increases from 250 to 310 °C. In terms of injection pressure, the interfacial energy increases rapidly from 126.46 to 245.68 kcal/mol, as shown Figure 7b. It can be concluded that the non-bonded interaction at the interface generates easily with applied injection pressure or elevated melting temperature. In other words, the increase in parameters above exerts a forcing effect on the entanglement of PA66 and PP molecules at the interface, which enhances the inter-diffusion of the two thermoplastic materials, and thus improves the interfacial energy significantly. However, as seen from Figure 7, when injection pressure or melting temperature increases to a certain extent, its resulting positive effect on the diffusion and non-bonded interaction of atoms at the interface will gradually decrease. In terms of injection pressure, it is likely that the stacking degree of polymer molecules at the interface is close to saturation at the pressure of 8 MPa. Furthermore, the relaxation of orientation of polymers at high melting temperature [34] may hinder the interaction between polymer molecules.

### 3.3. Unixial Tensile Deformation Process

**Figure 8:**
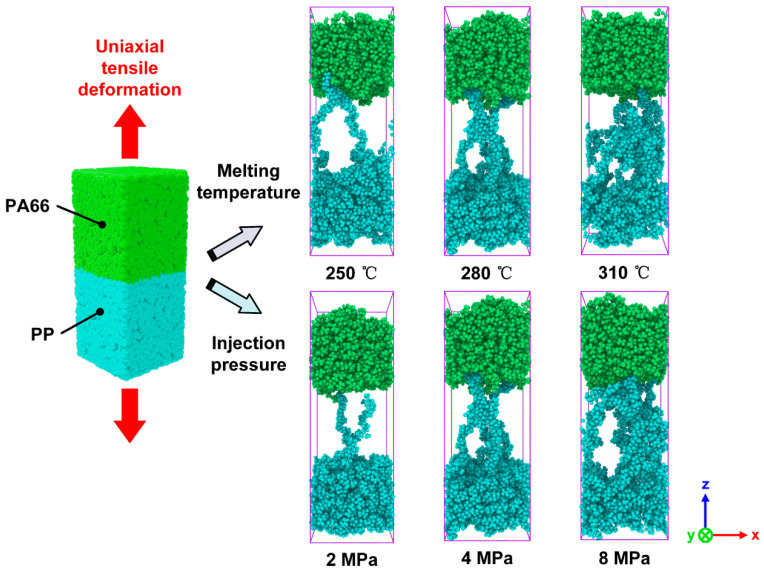
Snapshots of the damage modes in uniaxial tensile deformation of the combined PA66–PP interface under various processing parameters.

Uniaxial tensile deformation simulations were carried out to investigate the interfacial bonding strength and fracture mechanism of PA66–PP interface. Figure 8 shows the fracture morphologies of hybrid systems fabricated with various processing parameters. As can be seen, the failure process of the hybrid structure system is always accompanied by the pull-out of polymer chains, which is a typical behavior of thermoplastic materials [30]. The melting temperature has a weak effect on the failure mode of the PA66–PP interface when the injection pressure is fixed at 4 MPa. The failure mode is composed of adhesive failure and cohesive failure. However, more damage events in PP layer can be found at the melting temperature of 310 °C. On the other hand, when the melting temperature is set as 280 °C, the failure mode changes with injection pressure from 2 to 8 MPa. To be specific, at the lower injection pressure of 2 MPa, the failure mode is adhesive failure. With increasing injection pressure, the failure mode changes from the adhesive failure to the mixing mode of adhesive and cohesive failures. When the injection pressure increases to 8 MPa, the cohesive failure plays a predominant role, which indicates that the interface strength is higher than that of bulk polymers in this case. These findings suggest that there is a competition between the failure mechanism and enhancing effect due to the optimization of process parameters. The cohesive failure will be a primary damage event when the injection pressure or melting temperature can reach an optimum parameter.

**Figure 9:**
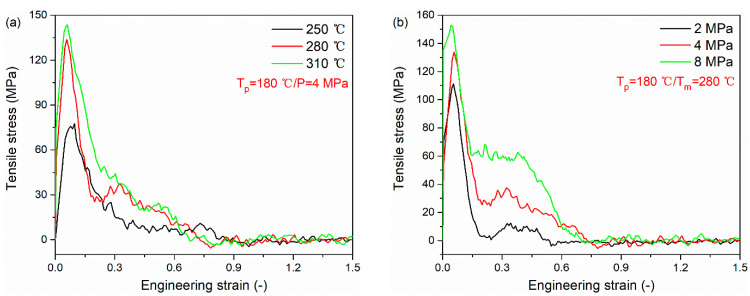
Tensile stress versus engineering strain curves for PA66–PP interface under various processing parameters: (**a**) melting temperatures and (**b**) injection pressure.

Figure 9 presents the tensile stress versus engineering strain curves for PA66–PP interface under various processing parameters. All curves are composed of two basic regions. At the initial stage, the curve increases linearly to the ultimate strength. After that, there is a softening region in which the tensile stress decreases slowly to zero. The tensile stresses of the PA66–PP interface at different melting temperatures and injection pressures are listed in Table 3. As can be seen, there is a positive correlation between the peak tensile stress and processing parameters (melting temperature and injection pressure). In particular, at the melting temperature of 280 °C, the peak tensile stress increases by 76.2% compared to that at the melting temperature of 250 °C. Meanwhile, further increasing melting temperature results in a weak increase in tensile stress. It can be understood that there is a limited processing temperature window. The same trend is also demonstrated in Figure 9b. At the injection pressure of 8 MPa, the peak tensile stress increase by 37.5% compared to that at the injection pressure of 2 MPa. Due to high injection pressure, the interface acquires excellent mechanical properties so that a greater energy is needed to destroy the interface. However, when the injection pressure is increased from 4 to 8 MPa, the peak stress shows a slower increment compared to the pressure increase from 2 to 4 MPa. In this case, the determinant of interfacial properties gradually changes from single pressure factor to multiple factors which interact on each other. The order of tensile stress varying in melting temperature and injection pressure is the same as that of interfacial bonding energy, validating the analysis about the results of uniaxial tensile deformation.

**Figure 10:**
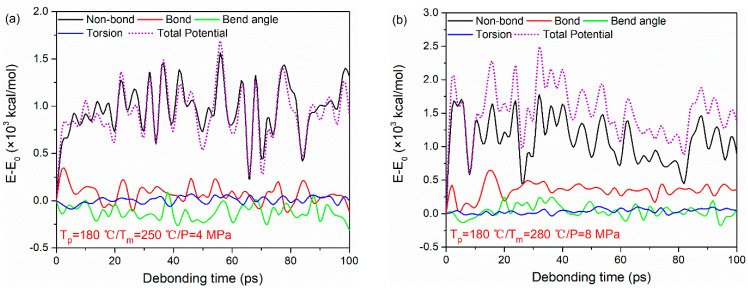
Potential energy decompositions of uniaxial tensile deformation process for PA66–PP interface under processing parameters: (**a**) *T*_p_ = 180 °C, *T*_m_ = 250 °C, *P* = 4 MPa and (**b**) *T*_p_ = 180 °C, *T*_m_ = 280 °C, *P* = 8 MPa.

The typical potential energy decompositions for the PA66–PP interface at *T*_p_ = 180 °C, *T*_m_ = 250 °C, *P* = 4 MPa and *T*_p_ = 180 °C, *T*_m_ = 280 °C, *P* = 8 MPa are taken as examples, as shown in Figure 10. As seen, the non-bonded interaction energy plays a crucial role during the tensile deformation of the PA66–PP interface regardless of processing parameters. The non-bonded interaction energy changes obviously with increasing strain. It increases sharply in the initial tensile loading, and then keeps at a relatively stable fluctuation range. Meanwhile, both bond stretching energy and angular bending energy experience an irregular change due to the elastic recovery of polymer molecules. However, there is a negligible change in dihedral energy with increasing strain. This behavior may be related to the failure mode of the system.

### 3.4. Sliding Deformation Process

**Figure 11:**
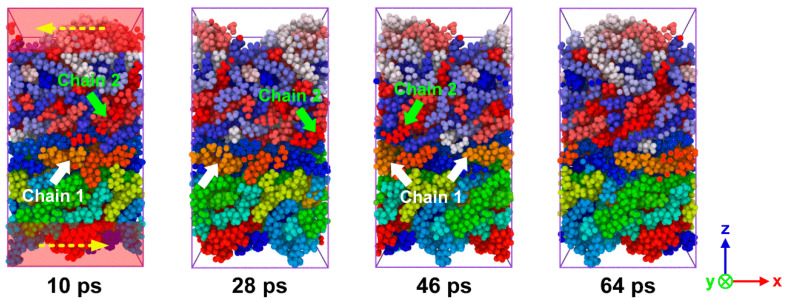
Snapshots of sliding process of the combined PA66–PP interface under *T*_p_ = 180 °C, *T*_m_ = 280 °C and *P* = 4 MPa.

In order to investigate the shear properties of the PA66–PP interface, the sliding deformation simulations were performed here. Figure 11 indicates the snapshots of sliding process of hybrid system fabricated at *T*_p_ = 180 °C, *T*_m_ = 280 °C and *P* = 4 MPa. To intuitively capture the morphological evolution of polymer molecules during the sliding process, all polymer chains were painted with different colors. The PA66 and PP chains move along the sliding direction separately, and then they cross the boundary and return to the system box from the opposite side due to the periodic boundary condition. When an external load is applied, the interface plays a role in the stress transfer between PA66 and PP layers. With further loading, the chains at the interface area gradually evolve from the curled state to the state of disentanglement. When the van der Waals interactions between the molecular chains were weak enough, the chains would be slipped because they could no longer resist external stress. Hence, the breakdown of non-bonded interactions between the PA66 and PP layer would result in the interfacial shear fracture.

Because of the boundary conditions, the failure morphologies of the composite system were not characterized by the damage events occurred at interface or polymer. Instead, the process is represented by unwinding and the continuous sliding motion of the molecular chains. Therefore, the stress–time curves do not show the classical trend in which displays in uniaxial tensile deformation or macro-experiments but exhibit a large fluctuation, as shown in Figure S2. The variations in shear stresses at PA66–PP interface with processing parameters under sliding loading are listed in Table 3. As illustrated in the above parts, the proper increase in melting temperature and injection pressure can significantly enhance the tensile stress of PA66–PP composite. However, unlike the tensile stress, the shear stress exhibits a slight decrease when the melting temperature increases from 280 to 310 °C. It is probably because of the loss of orientation of polymer chains at high temperatures as mentioned above. Meanwhile, the increase in the injection pressure could significantly increase the shear performance of the interface. It can be understood that a higher injection pressure leads to more intense penetrations of the polymer chains. Therefore, when the interface undergoes shear deformation, the tails of these interspersed chains would provide strong resistances with their physical structure similar to “pins”, resulting in a high shear stress.

The energy changes in various potential energy contributions at *T*_p_ = 180 °C, *T*_m_ = 250 °C, *P* = 4 MPa and *T*_p_ = 180 °C, *T*_m_ = 280 °C, *P* = 8 MPa are taken as examples, as shown in Figure S3. The sliding deformation is dominated by the non-bonded interactions instead of other compositions because the growth trend of non-bonded interaction is highly consistent with that of total potential energy. Although the general change trend of the curves is quite different from that of the uniaxial tensile deformation process, we can still get a clear conclusion that the non-bonded energy contributes mainly to the damage process of a PA66–PP heterogeneous interface subjected to both tensile and shearing loadings. In addition, the bond, bend angle and torsion energy experience a negligible change, illustrating that the bond breakage, angle and dihedral deformation are not involved during whole sliding process.

## 4. Conclusions

In the present study, MD simulations were firstly employed to study the bonding mechanism between SFR-PA66 and CFR-PP fabricated with different TCO processing parameters. After that, the interfacial behavior and fracture mechanism of hybrid composites under tensile and shear loadings were investigated. The main conclusions of this work are:

(1) During TCO process, the molecules from both PA66 and PP layers gradually accumulated near the final interface. A few molecules get across the interface, and tangle with the molecules from the other layer. Thus, it can be concluded that the interfacial bonding mechanism of hybrid composites is caused by molecules diffusion.
(2) Both melting temperature and injection pressure have a positive effect on the interfacial properties of PA66–PP interface. The diffusion coefficient and interfacial bonding energy of interface increase with the melting temperature and the injection pressure. Thus, a higher melting temperature and injection pressure lead to a higher tensile stress. While, the shear stress does not exhibit an obvious increase with increasing melting temperature because of the loss of orientation of polymer chains at high temperatures.
(3) Tensile failure modes of PA66–PP system are composed of adhesive failure and cohesive failure. The diffusion of polymer molecules is directly related to the interfacial failure. At higher melting temperature, more damage events in PP layers can be found because of strong interface. With increasing injection pressure, the failure mode changes from the adhesive failure to the mixing mode of adhesive and cohesive failures. The non-bonded interaction energy plays a crucial role during the tensile and sliding deformations of the PA66–PP interface.

## Figures and Tables

**Table 1:** Main parameters of polypropylene (PP) and polyamide 66 (PA66) atomistic models.

Material	Number of Chains	Degree of Polymerization	Total Amount of Atoms	Initial Density (g/cm^3^)	Box Size (nm^3^)
PP	16	50	7232	0.92	4.5 × 4.5 × 3.0
PA66	16	12	7328	1.14	4.5 × 4.5 × 3.1

**Table 2:** Main thermoplastic composite over-molding (TCO) processing parameters for short glass fiber reinforced polyamide 66 (SFR-PA66)/ continuous glass fiber reinforced polypropylene (CFR-PP) hybrid composites.

Serial Number	Melting Temperature (*T*_m_)	Injection Pressure (*P*)	Preheating Temperature (*T*_p_)
1	250 °C	4 MPa	180 °C
2	280 °C		
3	310 °C		
4	280 °C	2 MPa	
5		4 MPa	
6		8 MPa	

**Table 3:** Mechanical properties of PA66–PP interface under various TCO processing parameters.

Processing Parameters	250 °C/4 MPa	280 °C/4 MPa	310 °C/4 MPa	280 °C/2 MPa	280 °C/4 MPa	280 °C/8 MPa
Peak tensile stress (MPa)	75.9	133.7	143.8	111.2	133.7	152.9
Average shear stress (MPa)	15.1	53.0	52.9	46.9	53.0	75.8

## Supplementary Materials

The following are available online at https://www.mdpi.com/2073-4360/12/6/1270/s1, Figure S1: The MSD–time curve of PP and PA66 layer separately under various processing parameters: (**a**) melting temperatures and (**b**) injection pressure, Figure S2: Shear stress versus sliding time curves for PA66–PP interface under various processing parameters: (**a**) melting temperatures and (**b**) injection pressure, Figure S3: Potential energy decompositions of sliding deformation process for PA66–PP interface under processing parameters: (**a**) *T*_p_= 180 °C, *T*_m_= 250 °C, *P* = 4 MPa and (**b**) *T*_p_ = 180 °C, *T*_m_= 280 °C, *P* = 8 MPa.
