# Supplementary material for: Molecular Dynamics Simulation on the Interfacial Behavior of Over-Molded Hybrid Fiber Reinforced Thermoplastic Composites
Source: Polymers (Basel). 2020 Jun 2;12(6):1270. doi: 10.3390/polym12061270 (PMC7361982; doi:10.3390/polym12061270)
Supplement: Supplementary file 1 [file polymers-12-01270-s001.pdf]

polymers

MDPI

Supplementary Materials

Molecular Dynamics Simulation on the Interfacial Behavior of Over-molded Hybrid Fiber Reinforced Thermoplastic Composites

Bingyan Jiang [1,2], Muhan Zhang [1], Liang Fu [2], Mingyong Zhou [2] and Zhanyu Zhai [1,2,*]

[1] State Key Laboratory of High Performance and Complex Manufacturing, Light Alloy Research Institute, Central South University, Lushan South Road 932, Changsha 410083, China; jby@csu.edu.cn (B.J.); 183811004@csu.edu.cn (Mu.Z.)
[2] College of Mechanical and Electrical Engineering, Central South University, Lushan South Road 932, 410083 Changsha, China; fuliangcsu@csu.edu.cn (L.F); zhou1989@csu.edu.cn (Mi.Z.)
* Correspondence: zhanyuzhai@csu.edu.cn

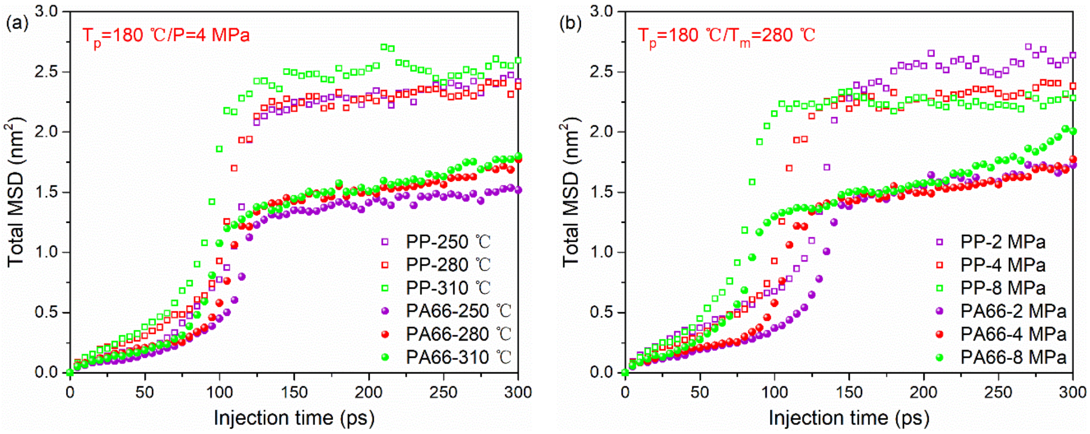

(**a**) (**b**)

Figure S1. The MSD–time curve of PP and PA66 layer separately under various processing parameters: (**a**) melting temperatures and (**b**) injection pressure.

The MSD–time curves of PP and PA66 layers during simulations under various processing parameters are given in **Figure S1**. As seen, the fluctuation trend of the curves of the two materials is basically consistent with the general trend of the whole system (**Figure 6** in the Article). The motions of PP molecules are significantly stronger than that of PA66 molecules due to their shorter chain length and lower stiffness.

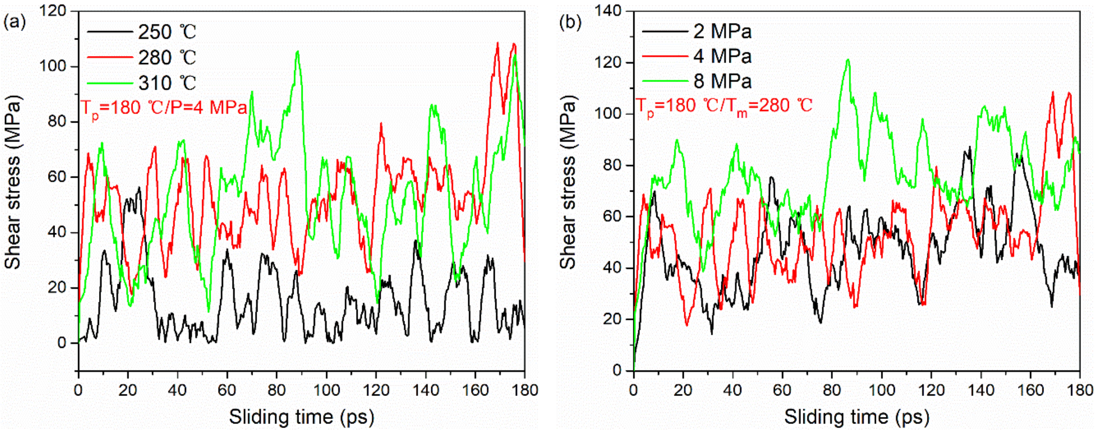

(**a**) (**b**)

Figure S2. Shear stress versus sliding time curves for PA66–PP interface under various processing parameters: (**a**) melting temperatures and (**b**) injection pressure.

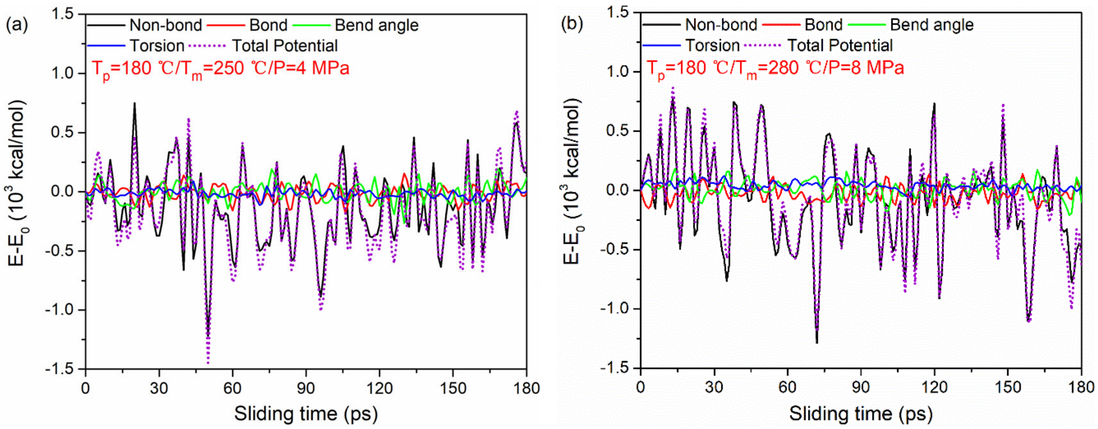

(**a**) (**b**)

Figure S3. Potential energy decompositions of sliding deformation process for PA66–PP interface under processing parameters: (**a**) T_p= 180 °C, T_m= 250 °C, P= 4 MPa and (**b**) T_p= 180 °C, T_m= 280 °C, P= 8 MPa.
